# Small Molecule-Induced Mitochondrial Disruption Directs Prostate Cancer Inhibition via Unfolded Protein Response Signaling

**DOI:** 10.18632/oncotarget.1130

**Published:** 2013-07-14

**Authors:** Elizabeth Rico-Bautista, Wenhong Zhu, Shinichi Kitada, Suthakar Ganapathy, Eric Lau, Stan Krajewski, Joel Ramirez, Jason A. Bush, Zhimin Yuan, Dieter A. Wolf

**Affiliations:** ^1^ Signal Transduction Program, Sanford-Burnham Medical Research Institute, La Jolla, CA; ^2^ NCI-designated Cancer Center, Sanford-Burnham Medical Research Institute, La Jolla, CA; ^3^ Proteomics Facility, Sanford-Burnham Medical Research Institute, La Jolla, CA; ^4^ San Diego Center for Systems Biology, Sanford-Burnham Medical Research Institute, La Jolla, CA; ^5^ Department of Radiation Oncology, University of Texas Health Science Center San Antonio, San Antonio, TX; ^6^ Biology Department, California State University, Fresno, CA; ^7^ Present address: Department of Genetics and Complex Diseases, Harvard School of Public Health, Boston, MA

**Keywords:** Prostate cancer, cell cycle arrest, apoptosis, oxidative stress, small molecule inhibitor, mitochondrial function

## Abstract

We previously identified SMIP004 (N-(4-butyl-2-methyl-phenyl) acetamide) as a novel inducer of cancer-cell selective apoptosis of human prostate cancer cells. SMIP004 decreased the levels of positive cell cycle regulators, upregulated cyclin-dependent kinase inhibitors, and resulted in G1 arrest, inhibition of colony formation in soft agar, and cell death. However, the mechanism of SMIP004-induced cancer cell selective apoptosis remained unknown. Here, we used chemical genomic and proteomic profiling to unravel a SMIP004-induced pro-apoptotic pathway, which initiates with disruption of mitochondrial respiration leading to oxidative stress. This, in turn, activates two pathways, one eliciting cell cycle arrest by rapidly targeting cyclin D1 for proteasomal degradation and driving the transcriptional downregulation of the androgen receptor, and a second pathway that activates pro-apoptotic signaling through MAPK activation downstream of the unfolded protein response (UPR). SMIP004 potently inhibits the growth of prostate and breast cancer xenografts in mice. Our data suggest that SMIP004, by inducing mitochondrial ROS formation, targets specific sensitivities of prostate cancer cells to redox and bioenergetic imbalances that can be exploited in cancer therapy.

## INTRODUCTION

Despite the considerable heterogeneity of clinical tumors [1], malignant transformation confers common characteristics to cancer cells – hallmarks - which include, among others, replicative immortality, resistance to negative growth signals and apoptosis, and the abilities to induce angiogenesis and to colonize distant organ sites [2]. Some of these phenotypic properties are now understood as results of positive selection of defined driver mutations in oncogenes and tumor suppressor genes [3, 4]. In prostate cancer, for example, such genetic drivers include loss of NKX3.1 and PTEN, amplification of Myc and the androgen receptor (AR), Sox9 [5], and fusions of *ETS* family transcription factors to androgen-inducible promoters [6]. Therapeutic targeting of driver mutations to which cancer cells have become ‘addicted’ has proven to be highly effective, with the BCR-ABL inhibitor imatinib representing a paradigm. However, many other oncogenic and tumor suppressive mutations remain difficult to target pharmacologically. In addition, molecular profiling by next generation sequencing is continuously adding further layers of complexity to the genetic make-up of cancer cells to complicate the development of broadly applicable targeted therapies [7].

Despite tumor heterogeneity, the expression of a common set of phenotypic properties, some of which also mark a common set of liabilities, can be exploited in therapy. The concept of “non-oncogene addiction” as introduced by Elledge and colleagues recognizes that cancer cells also rely on pathways that are not oncogenic per se but that nonetheless support their survival [8, 9]. Often as a direct result of oncogenic signaling, cancer cells are exposed to a series of stress conditions that are not ordinarily experienced by normal cells. This stress phenotype commands an increased reliance on rate-limiting stress support systems such as chaperones, DNA repair mechanisms, and antioxidant functions. Mechanisms of stress sensitization or stress overload that interact with the cancer phenotype in a synthetic lethal manner are consequently considered viable strategies for therapeutic intervention [9]. In fact, DNA damaging chemotherapeutics, hyperthermia and proteasome inhibitors already provide proof-of-principle for this approach.

The stress environment to which cancer cells are exposed typically includes a closely intertwined and mutually reinforcing network of oxidative, genotoxic, and proteotoxic stresses [9]. For example, intracellular accumulation of reactive oxygen species (ROS) through oncogenic signaling [10, 11] or as a result of hypoxia [12], sensitizes cancer cells to oxidative damage to DNA, proteins, and lipids. The production of ROS during mitochondrial respiration can be limited by the almost universally observed reprogramming of cancer cell metabolism toward aerobic glycolysis [13]. The increased reliance of cancer cells on glycolysis represents a requisite example of non-oncogene addiction that permits selective targeting by small molecules [14, 15].

In a high-content screen for compounds that upregulate the cyclin-dependent kinase inhibitor (CKI) p27^KIP1^, we previously identified *N*-(4-butyl-2-methylphenyl) acetamide (SMIP004) as a novel inducer of cancer cell-selective apoptosis of LNCaP human prostate cancer cells [16]. SMIP004 upregulated the CKIs p27 and p21 while decreasing the levels of positive cell cycle regulators, such as cyclin A, CDK4 and SKP2. The compound also suppressed intracellular CDK2 kinase activity, resulting in G1 arrest and inhibition of colony formation in soft agar. SMIP004 also induced cancer cell-selective apoptosis, but its mechanism of action remained unknown. We show here that SMIP004 has a novel activity in directing a mitochondrial pathway that leads to oxidative stress, activation of the unfolded protein response (UPR), and apoptosis.

## RESULTS

### SMIP004 Induces the Unfolded Protein Response

To assess global effects of SMIP004 at times when apoptosis has been initiated and to cross-reference these effects with those of other cytotoxic compounds, we obtained transcriptomic profiles of LNCaP-S14 cells exposed to SMIP004, to the DNA damaging agent camptothecin, to the proteasome inhibitor bortezomib (BTZ), and to the CDK inhibitor roscovitine for 24 h. Our LNCaP-S14 cell line, which was generated for the high-content screen that identified SMIP004, was derived by stably overexpressing the SKP2 subunit of the CRL1^SKP2^ ubiquitin ligase in human LNCaP prostate cancer cells. As a result of SKP2 overexpression, LNCaP-S14 cells exhibited marked downregulation of p27 and a reduced G1 population but behaved identically to parental LNCaP cells in terms of cellular responses to SMIP004 [16].

**Figure 1:**
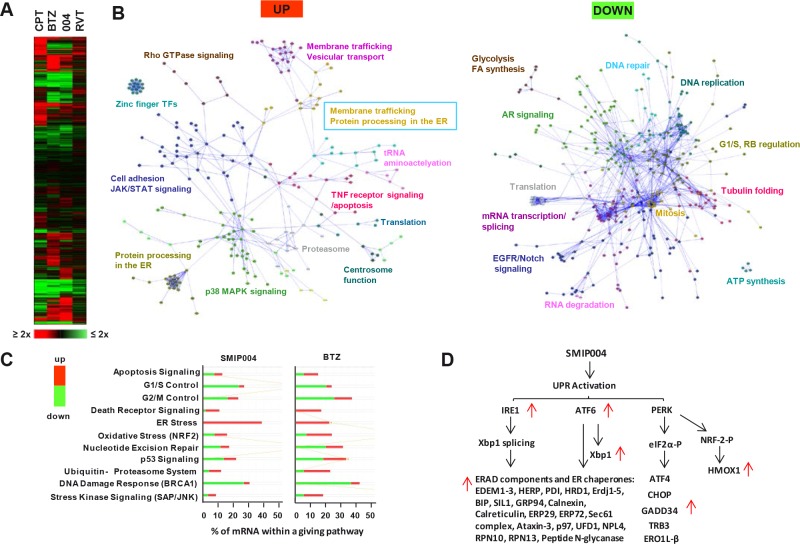
Effect of SMIP004 on global gene expression in LNCaP-S14 cells (A) LNCaP-S14 cells were treated with DMSO, SMIP004 (40 μM) or bortezomib (BTZ, 50 nM), camptothecin (CPT, 500 nM) or roscovitine (RVT, 20 μM) for 24 h. Total RNA was extracted and analyzed by expression microarray. Results are displayed as a clustered “heat map”. Upregulated transcripts are shown in red and downregulated transcripts in green. (B) Individual lists of SMIP004-induced and SMIP004-repressed mRNAs (±2-fold, p ≤ 0.05, derived from Supplementary Data File 3) were loaded into Cytoscape and used to build Reactome networks. The networks were clustered into modules, and pathways enriched in the modules (FDR ≤ 0.01) are indicated. Annotations highlighted by colored boxes refer to pathways represented by two different colors, those represented by the box frame and the font color. (C) Select IPA pathways that were enriched in the expression datasets. The x-axis indicates the percentage of mRNAs within a given pathway that are altered in response to SMIP004. (D) Transcripts affected by SMIP004 as a result of activation of the three arms of the UPR.

The SMIP004 mRNA expression signature was most similar to the signature induced by BTZ with a ~50% overlap in mRNAs changing more than 2-fold (p ≤ 0.05) (Fig. 1A) even though SMIP004 is not a proteasome inhibitor [16]. Network and pathway analyses revealed a general upregulation of stress and apoptotic pathways, including those involved in stress kinase, oxidative stress, death receptor and p53 signaling, and the ubiquitin-proteasome system (Fig. 1B, C). In addition, pathways involved in protein processing in the endoplasmic reticulum (ER) were strongly induced (Fig. 1B). This also included components of the ER stress-induced unfolded protein response (UPR) [17] such as *IRE1*, *ATF6*, and *XBP1*, and their transcriptional downstream targets (Fig. 1D). These findings suggested that SMIP004, like BTZ [18], acted as an UPR inducer.

**Figure 2:**
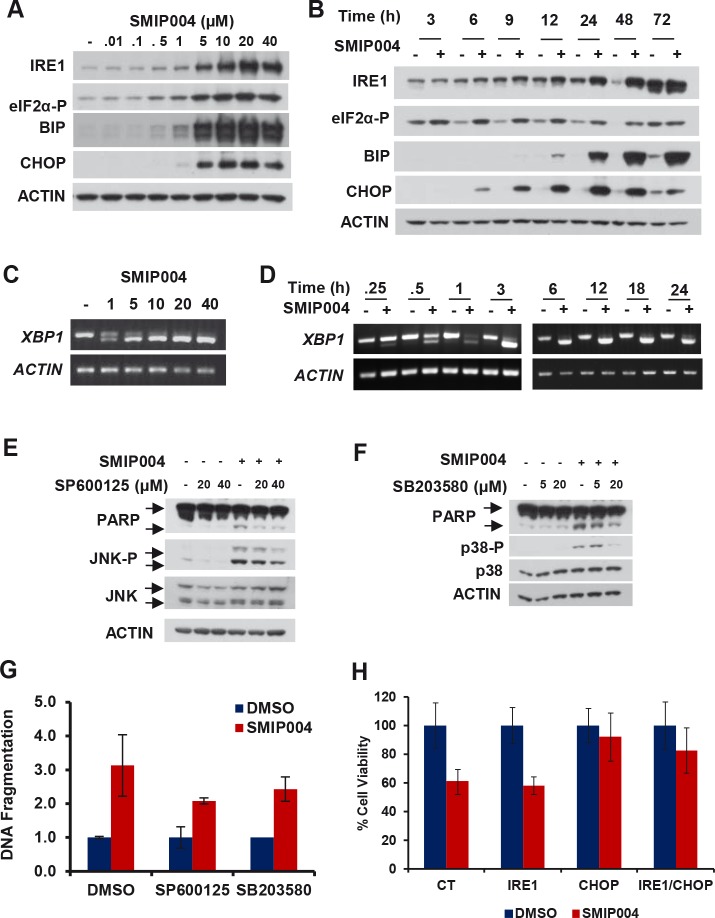
Effect of SMIP004 on the unfolded protein response (UPR) (A) Lysate from LNCaP-S14 cells treated with increasing concentrations of SMIP004 for 24 h were analyzed for UPR markers by immunoblotting. (B) Kinetics of UPR induction by 40 μM SMIP004. (C) XBP1 splicing was analyzed by RT-PCR with total RNA from LNCaP-S14 cells treated with increasing concentrations of SMIP004 for 24 h. (D) Kinetics of XBP1 splicing in response to 40 μM SMIP004. (E) Cells were treated with increasing concentrations of SMIP004 for 24 h in the presence of increasing concentrations of SP600125. JNK phosphorylation and PARP cleavage were analyzed by immunoblotting. (F) Cells were treated with increasing concentrations of SMIP004 for 24 h in the presence of increasing concentrations of SB203580. p38 phosphorylation and PARP cleavage were analyzed by immunoblotting. (G) DNA fragmentation was measured in extracts from cells treated with SP600125 (40 μM) or SB203580 (40 μM) and SMIP004 (40 μM) for 24 h. The graph represents the DNA fragmentation enrichment factor ± standard deviations (normalized to DMSO treatment) of three replicates. (H) Proliferation of cells transfected with specific siRNA for IRE1 and CHOP and treated with SMIP004 (10 μM) for 48 h was measured by MTT assay. The graph represents the mean ± standard deviations of four replicates.

SMIP004 caused rapid dose-dependent activation of all three arms of the UPR as indicated by phosphorylation of eIF2α (3 h) and upregulation of CHOP (6 h), IRE1 (9 h), and BIP (12 h) (Fig. 2A, B). α (3 h) and upregulation of CHOP (6 h), IRE1 (9 h), and BIP (12 h) (Fig. 2A, B). Splicing of transcription factor *XBP1* mRNA was induced at SMIP004 concentrations as low as 1 μM and as rapidly as 15 min after treatment (Fig. 2C, D). UPR induction was also observed in other prostate cancer cell lines, but not in normal human fibroblasts which do not die in response to SMIP004 (Fig. S1; [16]).

Prolonged ER stress can trigger two major pro-apoptotic pathways [19, 20]. Activated IRE1 promotes a cascade of phosphorylation events that culminates in the activation of JUN amino-terminal kinase (JNK) and p38 mitogen-activated protein kinase the sustained activation of which leads to cell death [21, 22]. Secondly, CHOP transcriptionally induces various pro-apoptotic factors [20]. We found that SMIP004 induced JNK and p38 activity (Fig. 2E, F), whereas chemical inhibitors of these kinases partially antagonized SMIP004-induced apoptosis (Fig. 2E - G). In addition, siRNA-mediated knockdown of IRE1 and CHOP rescued cell death induced by SMIP004 (Fig. 2H, see Fig. S1B for knockdown efficiencies). Thus, UPR signaling is the primary trigger of SMIP004-induced cancer cell apoptosis.

### SMIP004 Induces Destruction of Cyclin D1 via the Ubiquitin-Proteasome Pathway

**Figure 3:**
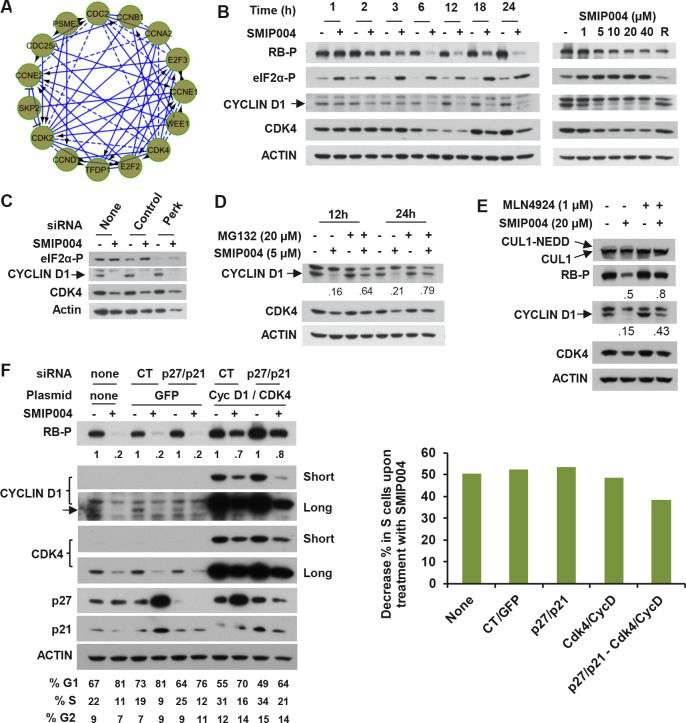
Effect of SMIP004 on cell cycle pathways (A) RB subnetwork components downregulated by SMIP004 as revealed by Reactome network analysis (B) Kinetics and dose response of SMIP004 effects on RB pathway components as revealed by immunoblotting. LNCaP-S14 cells were treated with 40 μM SMIP004 for the indicated times (left panel) or were treated for 24 h with the indicated doses of the compound (right panel). (C) LNCaP-S14 cells were transfected with siRNA for PERK, followed by treatment with DMSO or SMIP004 (40 μM). The levels of cyclin D1, CDK4 and eIF2α-P were determined by immunoblotting. (D) Cells were pre-incubated with MG132 for 1h followed by treatment with SMIP004 for the indicated times. Cyclin D1 and CDK4 levels were analyzed by immunoblotting. (E) Cells were pre-treated with the neddylation inhibitor MLN4924 (1 μM) for 1h followed by treatment with SMIP004 (20 μM) for 6h. Levels of cyclin D1, CDK4, RB-P and cullin 1 (neddylated and unneddylated forms) were evaluated by immunoblotting. (F) LNCaP-S14 cells were transfected with p27 and p21 siRNAs for 24 h followed by transient transfection with cyclin D1 and CDK4 expression plasmids for another 24 h. Cells were then incubated for an additional 24 h in the presence or absence of SMIP004 (40 μM). Cells were collected for flow cytometry and immunoblotting analysis. Quantifications of phospho-RB levels are relative to the loading control actin. Short and long exposures are shown for cyclin D1 and CDK4 blots. The right panel shows the effects of the various genes on SMIP004-induced decrease in the S phase population.

Before inducing apoptosis, SMIP004 arrests cell-cycle progression in G1 phase [16], but the mechanism of this arrest remained unidentified. Several ER stress-induced pathways have been implicated in G1 arrest, including those involving CHOP-dependent transcription [23], p27 accumulation through suppression of SKP2-mediated ubiquitylation [24], and inhibition of cyclin D1 mRNA translation via PERK-mediated phosphorylation of eIF2α [25, 26]. Downregulation of CHOP or p27 did not affect SMIP004-induced G1 arrest thus ruling out these pathways ([16] and data not shown). The transcriptomic analysis revealed marked downregulation of the RB tumor suppressor pathway (Fig. 1B) with major players such as cyclin D1, CDK4, and E2F mRNAs strongly suppressed (Fig. 3A). Consistently, cyclin D1/CDK4-mediated phosphorylation of RB on serine 780 was diminished in a dose-dependent manner beginning at 2 h after compound administration (Fig. 3B). This effect was closely paralleled by the rapid (~ 1h) downregulation and nuclear depletion of cyclin D1 protein (Fig. S2), whereas CDK4 levels only declined at later times (Fig. 3B). However, the inhibition of eIF2α phosphorylation by siRNA-mediated knockdown of PERK did not prevent SMIP004-mediated cyclin D1 downregulation (Fig. 3C), thereby implicating a mechanism that is distinct from those previously described [25-27].

Indeed, rapid cyclin D1 downregulation involved proteasomal degradation because it was efficiently antagonized by the proteasome inhibitor MG132 (Fig. 3D). In addition, consistent with the pervasive role of cullin-RING ubiquitin ligases (CRLs) [28] in cyclin D1 ubiquitylation, the NEDD8 E1 enzyme inhibitor MLN4924, which inactivate CRLs [29], substantially reversed SMIP004-mediated downregulation of cyclin D1 levels and RB phosphorylation (Fig. 3E). Knockdown of individual CRL substrate receptors implicated in cyclin D1 ubiquitylation (SKP2, FBXW8, FBXO31) did not rescue cyclin D1 degradation (data not shown), suggesting that SMIP004 can direct cyclin D1 degradation through multiple redundant pathways.

In an effort to determine whether the rapid inactivation of the RB pathway is required for SMIP004-induced cell cycle arrest, we sought to overcome the arrest by overexpressing cyclin D1 and CDK4. Overexpression of both cyclin D1 and CDK4 in untreated cells reduced the fraction of cells in G1 and increased the S phase fraction, indicating that their overexpression promoted G1-S progression (Fig. 3F). This combination also substantially rescued the inhibition of RB phosphorylation resulting from treatment of cells with SMIP004. Nevertheless, cell cycle arrest was not overcome by cyclin D1 and CDK4 overexpression even though the proteins were restored to levels considerably higher than those found in untreated cells (Fig. 3F). Apparently, in addition to cyclin D1/CDK4 downregulation, SMIP004 inhibits cell cycle progression through other mechanisms. We had previously shown that SMIP004 upregulates the CDK inhibitors p21 and p27, which might restrain the CDK activity required for cell-cycle progression downstream of RB [16]. Indeed, knockdown of p21 and p27 in cells overexpressing cyclin D1 and CDK4 partially prevented SMIP004-induced downregulation of the S phase population, suggesting that SMIP004 robustly impacts cell cycle progression at multiple levels.

### SMIP004 Induces UPR-independent Transcriptional Downregulation of the AR

**Figure 4:**
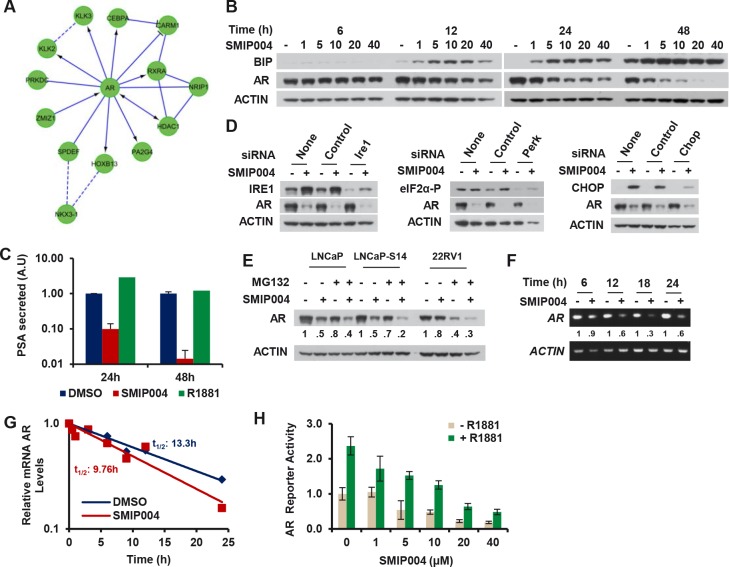
Effect of SMIP004 on androgen receptor levels (A) AR subnetwork components downregulated by SMIP004 as revealed by Reactome network analysis. (B) LNCaP-S14 cells were treated with increasing concentrations of SMIP004 (1 μM to 40 μM) and total cell extracts analyzed for BIP and AR by immunoblotting. (C) PSA secretion was measured in media from cells treated with SMIP004 (40 μM) and R1881 (0.2 nM) for the indicate times. (D) Cells were transfected with specific siRNA for IRE1, PERK and CHOP, and AR levels were analyzed by immunoblotting. (E) Parental LNCaP, LNCaP-S14 and 22RV1 cells were treated with the proteasome inhibitor MG132 (20 μM) and SMIP004 (40 μM) for 24 h. AR levels were evaluated by immunoblotting. (F) AR mRNA expression was evaluated by RT-PCR of total RNA isolated from cells treated with SMIP004 (40 μM) during the indicate time as described in Methods. (G) Quantitative real-time PCR was used to measure the stability of AR mRNA with and without SMIP004 (40 μM) in an actinomycin D chase experiment as described in Methods. (H) LNCaP-S14 cells were transfected with plasmids for AR-Luc and β-gal, followed by addition of increasing concentrations of SMIP004 for 24 h in the presence or absence of R1881 (0.2 nM). Luciferase activity was normalized to β-gal activity. The graph represents the mean ± standard deviations of three replicates.

The transcriptomic profile also indicated a suppression of AR signaling by SMIP004 (Fig. 1B) with several major AR target genes being strongly downregulated (e.g. KLK2, KLK3/PSA, HOXB13, NKX3-1; Fig. 4A). Due to the seminal importance of AR signaling in prostate cancer which is maintained even after it has progressed to the castration resistant state [30], we sought a deeper understanding of the antiandrogenic activity of SMIP004. The compound induced downregulation of the AR protein in a manner that closely paralleled UPR induction in terms of timing and dose dependence as determined by BIP accumulation (Fig. 4B). SMIP004-induced AR downregulation was functionally consequential because it drastically suppressed the secretion of PSA (Fig. 4C).

UPR signaling induced by the SERCA inhibitor thapsigargin was previously shown to inhibit the synthesis of the AR protein, possible due to inhibitory phosphorylation of eIF2α [31]. However, siRNA-mediated knockdown of PERK, IRE1, or CHOP did not impair SMIP004-mediated AR downregulation (Fig. 4D), suggesting that it occurs in a UPR-independent manner. Induced proteolysis via ubiquitin-proteasome system is a major mode of AR downregulation by HSP90 inhibitors [32, 33] and AhR agonists [34]. In contrast, SMIP004-induced AR downregulation was not abrogated by the proteasome inhibitor MG132 either in LNCaP-S14 or parental LNCaP cells or in another AR positive prostate cancer cell line, 22RV1 (Fig. 4E). Instead, RT-PCR analysis indicated that SMIP004 downregulated AR mRNA (Fig. 4F). Since AR mRNA stability was only modestly affected by SMIP004 (t_1/2_ = 9.76 h; Fig. 4G) versus DMSO (t_1/2_ = 13.3 h), we performed reporter gene assays to determine the impact of SMIP004 on AR promoter activity. AR promoter activity was repressed by SMIP004 in a dose-dependent manner (Fig. 4H). AR agonist-induced activity was likewise suppressed (Fig. 4H). In summary, these data suggest that SMIP004 abrogates AR signaling primarily through UPR-independent transcriptional downregulation of AR expression.

### SMIP004 Induces Mitochondrial Disruption and Oxidative Stress

**Figure 5:**
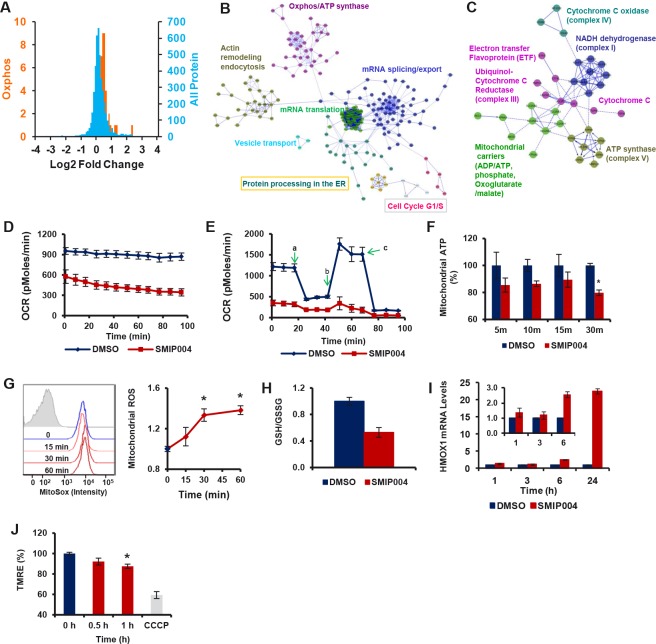
Effect of SMIP004 on protein expression and metabolism (A) The blue curve shows relative ratios of all 4421 proteins quantified by SILAC in cells treated with 40 μM SMIP004 for 3 h. 92.4% of all proteins showed a log2 ratio of ~0. The orange curve shows the ratios of 36 proteins involved in oxidative phosphorylation (Oxphos), which are significantly higher than 0. The same 36 proteins are shown in (C). (B) Reactome network built from proteins significantly upregulated by SMIP004. The network was clustered into modules, and pathways enriched in the modules (FDR ≤ 0.01) are indicated. Annotations highlighted by colored boxes refer to pathways represented by two different colors, i.e. those represented by the box frame and the font color. (C) Oxphos subnetwork clustered into individual complexes of the electron transport chain. (D) Basal oxygen consumption rate (OCR) in the presence or absence of SMIP004 in LNCaP-S14 cells was evaluated with the XF-24 Extracellular Flux Analyzer as described in Experimental Procedures. The graph represents average OCR ± SEM of three replicates. The measurements were begun (0 min time point) 10 minutes after adding vehicle or SMIP004 (40 μM) to the cells. (E) Mitochondrial respiration profile of LNCaP-S14 cells. Cells were pre-incubated for 2h with vehicle (DMSO) or SMIP004 (40 μM), followed by real-time analysis of cellular respiration in an XF24. Oligomycin (a) was added to inhibit ATP synthase, FCCP (b) to uncouple respiration from ATP synthesis, and rotenone (c) to inhibit complex I and mitochondrial respiration. The graph represents the mean oxygen consumption rate (OCR) ± SEM of three replicates. (F) LNCaP-S14 cells were treated with SMIP004 (40 μM) or DMSO in the presence of 2-deoxyglucose (2-DG) for the indicated time. 2-DG (10 mM) was added 30 min before SMIP004 to inhibit glycolytic ATP production. ATP levels were measured using the ApoSENSOR™ ATP Assay kit (BioVision). The graph represents the average ± SEM of eight replicates. * p-value: 0.00007. (G) Cells were treated with SMIP004 (40 μM) for 15, 30 or 60 minutes and mitochondrial superoxide was measured as described in Methods. The figure shows representative histograms of flow cytometry experiments demonstrating an increase in mean fluorescence intensity of oxidized MitoSox following SMIP004 treatment (filled grey histogram represents unstained cells). The graph represents the mean fold-induction ± SEM of nine replicates from three independent experiments. * p-value: 0.0001. (H) Cells were treated with SMIP004 (40 μM) for 6h and intracellular levels of reduced (GSH) and oxidized glutathione (GSSG) were determined as described in Methods. The graph shows the mean GSH/GSSG ratio ± standard deviations of three replicates. (I) HMOX1 mRNA levels were determined by qPCR in total RNA from cells treated with SMIP004 (40 μM) for the indicated time points. The graphs represent the means ± standard deviations of two independent experiments, each performed in duplicates. Data were normalized to DMSO. The inset shows blow-ups of the 1, 3, and 6h time points. (J) Cells were treated with SMIP004 (40 μM) for the indicated time points and mitochondrial membrane potential was measured as described in Methods. The graph represents the means ± SEM of two independent experiments, each performed in triplicates. * p-value: 0.0021. The mitochondrial uncoupler CCCP was used as positive control.

To gain insight into proximal mechanisms determining the pleiotropic cellular activities of SMIP004, we performed a SILAC experiment [35] to obtain a quantitative proteomic profile of the response of prostate cancer cells to short-term exposure to SMIP004 (3h). Whereas the vast majority of the ~4500 proteins measured retained a log2 ratio very close to 0 in SMIP004 treated versus untreated cells (Fig. 5A, Supplementary Data File 1), SMIP004 induced the upregulation of 580 proteins and the downregulation of 168 proteins (1.2-fold, p ≤ 0.05). Network analysis revealed induction of biosynthetic pathways by SMIP004, which included mRNA splicing, export and translation, protein processing in the endoplasmic reticulum (ER), and vesicular transport (Fig. 5B, Fig. S3A). SMIP004 also upregulated 110 proteins belonging to the Gene Ontology terms Mitochondrial Matrix and Mitochondrial Membrane. Of these, 36 are directly involved in oxidative phosphorylation, including members of the electron transport chain (ETC), ATP synthase, and several mitochondrial carriers of the SLC25 family (Fig. 5A, C).

The proteomic profile suggested that SMIP004 altered mitochondrial activity, potentially increasing the flux through the respiration chain. Surprisingly, we found that SMIP004 led to a rapid and pronounced reduction in basal and maximal (uncoupled) mitochondrial oxygen consumption (Fig. 5D, E; Fig. S3B). The compound also reduced the cellular ATP derived from mitochondria, although levels rebounded after 1 – 2 h (Fig 5F, Fig. S3C).

Since ETC inhibition is a well-known mechanism of mitochondrial ROS formation [36, 37], we asked whether SMIP004 caused oxidative stress. Indeed, SMIP004 led to increased mitochondrial ROS formation beginning as early as 30 minutes (Fig. 5G, Fig. S3D). In addition, the compound caused a decrease in the ratio of reduced to oxidized glutathione (Fig 5H) and a time-dependent increase in the expression of hemeoxygenase 1 (HMOX1) mRNA (Fig 5I), a prominent target of the oxidative stress-inducible transcription factor NRF2 [38]. Finally, SMIP004 led to a mild reduction in the mitochondrial membrane potential (Fig. 5J), a finding that is consistent with the local toxicity of increased ROS production [39].

**Figure 6:**
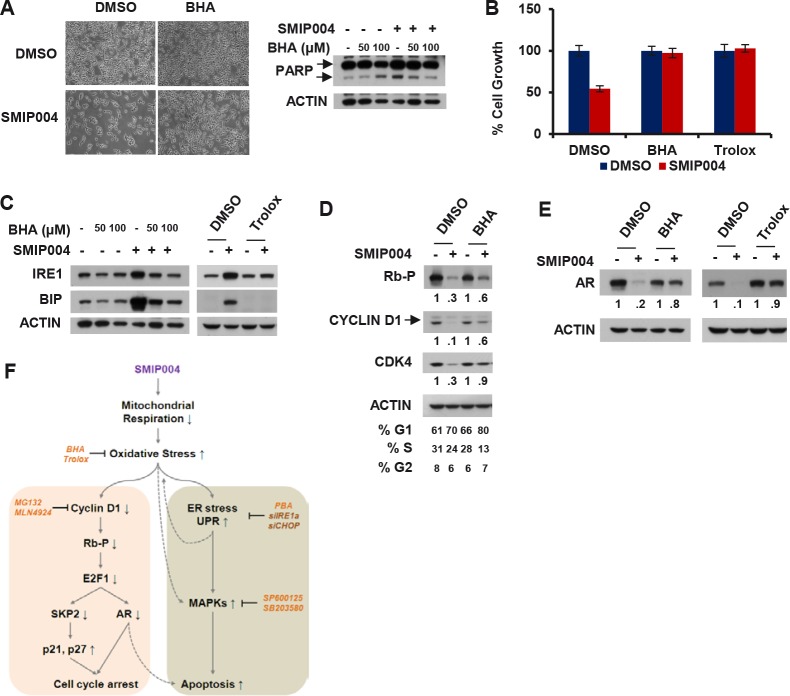
Effect of antioxidants on SMIP004-mediated cellular responses (A) LNCaP-S14 cells were treated with vehicle (DMSO) or SMIP004 (40 μM) for 24 h in the presence or absence of butylated hydroxyanisole (BHA, 100 μM). Cells were examined by phase contrast microscopy prior to cell lysate preparation for analysis of PARP cleavage. (B) Cell growth was analyzed by MTT assay in LNCaP-S14 cells treated with SMIP004 (40 μM) for 24 h in the presence or absence of BHA (100 μM) and trolox (2 mM). The graph represents the means ± standard deviations of at least two independent experiments, each performed in triplicates. (C) Total lysate from cells treated with SMIP004 for 24 h in the presence or absence of BHA or trolox was analyzed for UPR markers. (D) Effect of antioxidants on cell cycle regulators. (E) Effect of antioxidants on AR levels. (F) Diagram summarizing SMIP004 actions. Stippled lines indicate putative effects that were not experimentally addressed in the present study. BHA, butylated hydroxyanisole (antioxidant); trolox, antioxidant; MG132, proteasome inhibitor; MLN4924, Nedd8 activating enzyme inhibitor; PBA, 4-phenylbutyric acid (chemical chaperone); SP600125, JNK Inhibitor II; SB203580, p38 inhibitor.

Consistent with the above results, a screen for chemical compounds able to antagonize the cytotoxic activity of SMIP004 identified the ROS scavengers butylated hydroxyanisole (BHA) and trolox, which potently rescued cells from SMIP004-induced apoptotic cell death (Fig. 6A, B, S4). BHA and trolox also abrogated all other cellular effects of SMIP004, including UPR induction (Fig. 6C), inhibition of RB phosphorylation and cyclin D1 proteolysis (Fig. 6D), and transcriptional downregulation of the AR (Fig. 6E). These findings place SMIP004-induced oxidative stress on top of a cascade that culminates in cell cycle arrest and cancer cell selective apoptosis (Fig. 6F and Discussion).

### SMIP004 Inhibits Prostate Cancer Cell Growth in Vivo

**Figure 7:**
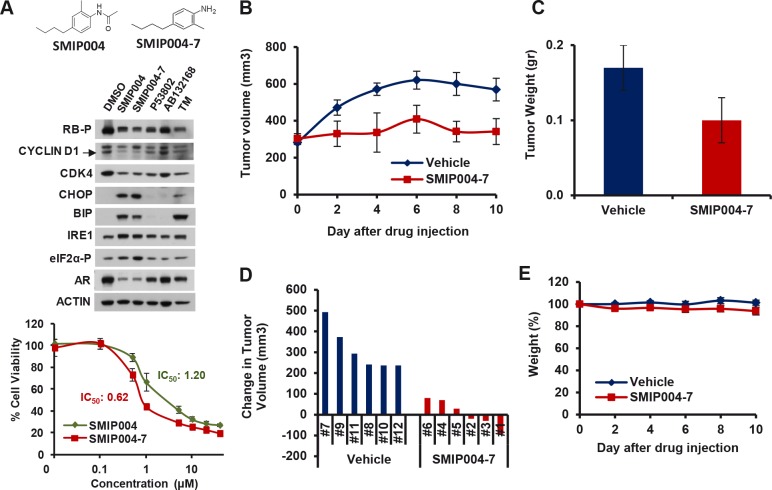
Effect of a SMIP004 analog on tumor growth in vivo (A) The chemical structures of parent compound SMIP004 and its analog SMIP004-7 are shown in the top panel. The middle panel shows the effect of several SMIP004 analogs on UPR activation and cell cycle regulators. LNCaP-S14 cells were treated with DMSO (0.1%), SMIP004 (40 μM), SMIP004-7 (40 μM), or the inactive analogs P53802 (40 μM), AB132168 (40 μM) for 24 h. UPR activation and cell cycle regulators were analyzed by immunoblotting. The glycosylation inhibitor tunicamycin (TM, 1 μM) was used as a positive control for UPR induction. Cell viability was measured by MTT assay in cells treated with SMIP004 or SMIP004-7 (lower panel). The graph represents the mean of four replicates ± standard deviations. (B) LNCaP-S14 xenografts were grown in SCID mice. Six animals received SMIP004-7 (50 mg/kg i.p.) for 10 days while the remaining six mice were treated with vehicle. The graph represents mean tumor volumes ± standard deviations in each group over time. (C) Final weights of tumors excised from mice treated with vehicle or SMIP004-7. The graph represents the average of three mice ± standard deviations. (D) The response to SMIP004-7 or vehicle for individual animals was expressed as change in tumor volume (i.e. day 9 minus day 0). (E) The graph represents relative average body weights ± standard deviations in the SMIP00-7 treatment and DMSO control groups.

Given its robust modulation of several cellular pathways that are highly relevant to prostate cancer such as AR signaling and the cyclin D1/CDK4/RB axis [40], we tested whether SMIP004 exhibited anti-prostate cancer activity in murine xenograft models. For these studies, we used the more potent analog SMIP004-7 (Fig. 7A). The analog evoked identical cellular pathways as the parental compound but had a twofold lower IC_50_ (620 nM, Fig. 7A). We first determined the maximally tolerated dose of SMIP004-7 (~200 mg/kg intraperitonially, i.p.; data not shown). SCID mice bearing LNCaP-S14 tumors were dosed with 50 mg/kg i.p. daily. SMIP004-7 potently suppressed tumor growth as judged by average tumor volumes (Fig. 7B). The final tumor weights were ~40% lower in SMIP004-7 treated animals (average 340 ± 70 mm^3^, 0.10 ± 0.03g, Fig. 7C) compared to those of the vehicle control group (average 570 ± 61 mm^3^, 0.17 ± 0.03 g; Fig. 7C). SMIP004-7 completely inhibited tumor growth in three of six animals (#1, 2 and 3) and greatly retarded tumor growth in the remaining three mice, while vehicle control mice showed an increase in tumor volume of at least 250 mm^3^ (Fig. 7D). Only insignificant weight loss was observed (Fig. 7E). Blood chemistry and histology of major organs (heart, lung, spleen, pancreas, gut and skeletal muscle), were largely normal at the end of the treatment period (Table S1). Occasional splenomegaly was, however, observed (data not shown). In addition, some hepatocytes of treated mice displayed increased nuclear size and multinucleation (Fig. S5A). These phenotypes resemble those of SKP2^−/−^ knockout animals [41], and the finding is therefore consistent with the known activity of SMIP004 to downregulate SKP2 [16].

To rule out that the reduction in tumor growth was due to events other than the administration of SMIP004-7, we suspended compound treatment of 3 mice for 17 days. During the suspension period, the residual tumors resumed growth indicating that the cells originally received by these animals were competent of forming tumors. When tumors reached ~500 mm^3^, the pretreated animals were subjected to a second round of treatment with an increased dose of SMIP007-4 (100 mg/kg daily) for 10 days. As in the first treatment period, SMIP004-7 receiving animals showed a strong inhibition in tumor growth whereas tumors grew to large sizes in vehicle treated mice (Fig. S5B). These results were confirmed by final average tumor weights and by individual changes in tumor volumes, whereas no significant weight loss was observed (Fig. S5C – E). These data firmly suggest that pretreated animals bore growth competent tumors and that the observed tumor inhibition is thus due to the pharmacological activity of SMIP004-7.

To further validate the anti-tumor activity of SMIP004-7, we performed further xenograft studies using an additional AR-positive human prostate cancer cell line, LAPC4. SMIP004-7 elicited the same effects on LAPC4 as on LNCaP-S14 cells in terms of growth inhibition, downregulation of RB phosphorylation and AR expression, and UPR activation (Fig. S5F), but was ~5 times more potent than the parent compound SMIP004 in inducing cell death (IC_50_ = 0.68 vs. 3.53 μM, Fig. S5G). Animals with similar initial LAPC4 tumor burden were paired and assigned to either the drug or vehicle cohort. SMIP004-7 exhibited profound tumor inhibitory activity in this system with 5 out of 6 animals displaying lower tumor volumes upon treatment for 7 days (Fig. S5H).

Lastly, we found that SMIP004-7 tumor inhibitory activity was also observed in the MDA MB-231 breast cancer xenograft model. Treatment with SMIP004-7 for three weeks inhibited tumor growth ~5-fold without any changes in body weight (Fig. S5I), suggesting that SMIP004-7 may have broader anti-cancer activity.

## DISCUSSION

### Mechanisms of SMIP004-Directed Cell Cycle Arrest and Apoptosis

Our combined chemical genomic and proteomic profiling followed by functional validation of predicted pathways revealed a first model for the mechanism of action of SMIP004 (Fig. 6F). According to this scheme, SMIP004 induces oxidative stress, which then triggers independent pathways that drive cell cycle arrest and apoptosis.

Proteolytic downregulation of cyclin D1 likely initiates the cell cycle arrest because it commences within ~2 hours after SMIP004 addition. Proteolysis involves the ubiquitin-proteasome pathway and members of the CRL family of E3 ubiquitin ligases, although the exact identity of the E3(s) remains to be established. Regardless, the SMIP004-induced proteolytic mechanism is very potent as it clears a large portion of cyclin D1, even when it is strongly overexpressed (Fig. 3F). The compound might therefore be particularly effective against the abundant class of malignancies driven by high levels of cyclin D1 [42]. Suppression of cyclin D1/CDK4 activity would result in reduced RB phosphorylation and E2F transcription factor activity [43]. As a result, E2F target genes such as SKP2 will be suppressed [44], a prediction we have confirmed previously [16]. The downregulation of SKP2 would then stabilize p21 and p27 to reinforce the G1 cell cycle arrest.

Like cyclin D1, AR is downregulated by SMIP004, albeit with slower kinetics and through a distinct mechanism involving transcriptional repression. E2F1 is a direct activator of the AR promoter, and loss of RB is associated with increased AR levels and progression of prostate cancers to castration resistance [40]. The suppressive effects of SMIP004 on the cyclin D1/RB/E2F pathway may therefore underlie transcriptional AR downregulation (Fig. 6F). Irrespective of the exact mechanism, SMIP004-induced AR downregulation is significant for advanced prostate cancer, which typically maintains dependence on the AR protein even after progression to castration resistance [30]. Whereas AR downregulation may augment SMIP004-induced cell death, we do not regard it as a primary trigger because of its delayed onset (12 – 24 h).

Rather, based on kinetics, siRNA, and chemical inhibitor studies, UPR signaling resulting in MAPK activation was identified as the primary pathway executing SMIP004-induced cancer cell apoptosis. UPR signaling is principally geared towards cell survival but induces apoptosis upon severe insult [19]. Many human cancers display a chronically induced UPR, potentially as adaptation to microenvironmental stresses [45]. However, with regards to the well recognized “stress phenotype” of cancer cells [9], such a survival strategy can turn maladaptive when confronted with therapeutic regimens that exhaust the stress support network. We propose that SMIP004 is such a regimen, and that the cancer cell selectivity of the compound derives from the differential sensitivity to proapoptotic UPR signaling.

### Mitochondrial Oxidative Stress as a Proximal Element of the Mechanism of Action of SMIP004

Several lines of evidence indicate that SMIP004 directly induced a pathway leading to oxidative stress: (i.) The compound increased mitochondrial ROS within 15 minutes. (ii.) All cellular effects of SMIP004 were rescued by two different antioxidants. (iii.) SMIP004 caused a rapid decrease in the GSH/GSSG ratio. (iv.) The compound led to induction of the NRF2 transcriptional pathway, in particular the mRNA for HMOX1 (Fig. 5G).

Notably, a relatively small increase in mitochondrial ROS (~40%) led to a dramatic decrease in cell viability. This finding highlights the low tolerance of cancer cells for extraneous stressors, presumably because their endogenous stress defense mechanisms are already stretched to the limits. Consistently, we noticed that LNCaP-S14 cells display very high basal levels of ROS relative to primary human fibroblasts (data not shown). Presumably as an adaptive measure, NRF2 is already localized in the nucleus and thus activated in untreated LNCaP-S14 cells (Fig. S3E). While SMIP004 caused an additional activation of the NRF2 transcriptional program geared toward stress defense (GCLM, X^−^_C_ system, NQO, TRXR1, HMOX1, Supplementary Data File 2), compound exposed cells could no longer capitalize on the pro-survival function of this program [46]. This may be due to paradoxical SMIP004-mediated downregulation of prominent NRF2 target genes involved in glutathione utilization and regeneration (GLRX, GSR, GPX1, 2, 4, most glutathione-S-transferases, Supplementary Data File 2). Whereas it remains unclear how SMIP004 promotes the selective activation of NRF2 target genes, this imbalance may contribute to the observed decrease in the GSH-GSSG ratio and the lethal consequences of oxidative stress.

The mechanism of ROS-induced apoptosis are not well understood [47] but UPR induction is a known consequence of oxidative stress [48]. ROS can cause protein misfolding in the ER through oxidation of amino acids or by perturbing calcium-dependent ER chaperone activity. Conversely, ER stress can trigger oxidative stress through CHOP-mediated induction of ERO1-like enzymes, which release H_2_O_2_ during protein disulfide isomerase-catalyzed formation of disulfide bonds. ERO1-LB mRNA was upregulated ~3.5-fold by SMIP004. Since the ER is a highly oxidizing environment, it is particularly susceptible to oxidative stress and the vicious cycle resulting from it. Cancer cells originating from secretory tissues with high traffic through the ER, such as prostate epithelial cells, are expected to be preferentially sensitive to such conditions.

### Potential Utility of Disruptors of Mitochondrial Redox Balance for Cancer Therapy

Insights gained over the past 15 years have put the concept of pharmacological targeting of the increased oxidative stress sensitivity of cancer cells on a firm foundation [9, 47, 49, 50]. Several high profile compounds, including erastin [51], piperlongumine [52], and elesclomol [53] have emerged, which cause ROS-dependent cell death selectively in cancer cells. While mechanisms and targets vary, some of these compounds are already being tested in the clinic.

The molecular target(s) of SMIP004 remain to be identified. Based on the instant kinetics with which the compound inhibits mitochondrial ATP production and stimulates mitochondrial ROS (5 – 30 minutes), it is tempting to speculate that SMIP004 directly interferes with the ETC. SMIP004 may therefore have a similar mechanism of action as the chelating agent elesclomol. This compound does not appear to have a protein target per se but rather to interfere more broadly with the mitochondrial electron transport process through copper-dependent redox cycling [54]. The coordinated upregulation of ETC components observed in our proteomic profile (3 h) may therefore reflect a compensatory but ultimately futile effort of SMIP004 exposed cells to uphold electron transport. Interestingly, a yeast screen for elesclomol sensitive heterozygous diploids identified a network of mitochondrial and protein anabolic processes that largely overlapped with that we obtained through proteomic profiling [54].

In the era of “targeted therapy” and “personalized health care”, there remain questions as to the viability of pleiotropic stress response pathways as cancer drug targets. The following points may be considered: (i.) Many clinically efficacious cancer drugs, including bortezomib, cisplatin, and etoposide, rely on ROS formation for efficacy [47, 49]. (ii.) Just like redox disruptors, a surprising number of “targeted” drugs in advanced stages of clinical testing, including proteasome, MAPK, and mTOR inhibitors, target molecules that are essential to the survival of normal cells and thus not per se cancer cell selective. Here, cancer selectivity appears to come from the phenomenon of non-oncogene addiction [9]. (iii.) Redox active therapeutics are not necessarily incompatible with the emerging concept of personalized medicine. To the contrary, they stand to benefit from the development of biomarkers just as much as any other form of therapy. In the future, detailed redox pheno- and genotyping could guide the selection of specific drugs such as SMIP004 that efficiently target the redox vulnerability of individual cancers.

## Methods

### Chemical Compounds

SMIP004 was purchased from Ryan Scientific and verified by HPLC-MS. SMIP004-7 is an active analog of SMIP004 purchased from Maybridge Ltd. MG312 was from Boston Biochem, bortezomib (BTZ) from LC Laboratories, camptothecin (CPT) and butylated hydroxyanisole (BHA) from Sigma-Aldrich. Roscovitine (RVT) was from Biomol International, SP600125 from EMD Chemicals Inc., SB203580 and tunicamycin from Enzo Life Sciences, and trolox from Santa Cruz Biotechnology. Actinomycin D was from MP Biomedicals, 2-deoxy-glucose from Tokyo Chemical Industry (TCI) and cycloheximide from Acros Organics. MLN4924 was a gift from M. Petroski. FCCP, oligomycin and rotenone were a gift from V. Kodali. Drugs were dissolved in DMSO (unless specified otherwise in the product data sheet) and kept at −20°C.

### Antibodies

Antibodies directed against IRE1α (3294, 1:1000), BIP (3177, 1:2000), CHOP (2895, 1:1000), phospho-eIF2α (Ser 51) (9721, 1:1000), cyclin D1 (2922, 1:1000, for immunoblotting), PARP (9542, 1:1000), phospho-SAPK/JNK (4668, 1:1000), SAPK/JNK (9252, 1:1000), p38 (9212, 1:1000), phospho-p38 (9216, 1:500), phospho-Rb (Ser780) (9307, 1:2000) were obtained from Cell Signaling Technology. Antibodies recognizing CDK4 (sc-260, 1:5000), cyclin D1 (A-12) (sc-8396, for immunofluorescence 1:250), AR (441) (sc-7305 1:1000) and NRF2 (C-20) (sc-722 1:200 for immunofluorescence) were from Santa Cruz Biotechnology. Antibodies against CUL1 (32-2400, 1:1000) were from Zymed Laboratories and anti-actin (69100, 1:5000) was from MP Biomedicals, LLC. HRP Donkey anti-mouse IgG (715-035-150, 1:5000) and HRP Donkey anti-rabbit IgG (711-035-152, 1:5000) were from Jackson ImmunoResearch Laboratories.

### Tissue Culture

LNCaP-S14 cells were obtained by stably overexpressing SKP2 in LNCaP cells as described previously [16]. LNCaP-S14 and other prostate cancer cells (LNCaP, PC3 and DU145) were maintained in RPMI supplemented with 10% fetal bovine serum and 5 % penicillin/streptomycin solution. IMR90 cells were maintained in DMEM supplemented with 10% fetal bovine serum, 5% penicillin/streptomycin and 4 mM glutamine. 22RV1 cells were maintained in RPMI with high glucose (4.5 mg/ml). LAPC4 cells were maintained in Iscove's Modified Dulbecco's Media (IMDM). Cells were grown as a monolayer in a humidified incubator at 37°C and 5% CO_2_. For experiments with LNCaP-S14 cells, cells were plated in poly-lysine coated plates one day before treatments. Cell viability was assessed using the 3-(4,5-dimethylthiazol)-2,5-diphenyl tetrazolium bromide (MTT) cell proliferation assay (ATCC^®^). Cell cycle analysis was done by flow cytometry as described previously [16] at the Flow Cytometry Facility of the Sanford Burnham Medical Research Institute. Apoptosis was measured using the Cell Death Detection ELISA (Roche) and by immunoblotting for PARP cleavage.

**Table:** 

Primers			
Regular PCR	Forward 5' → 3'	Reverse 5' → 3'	Ref
XBP1	CCTTGTAGTTGAGAACCAGG	GGGGCTTGGTATATATGTGG	[55]
AR	CGGAAGCTGAAGAAACTTGG	CGTGTCCAGCACACACACA	[31]
ACTIN	GGTCAGGATCTTCATGAGGT	TCTACAATGAGCTGCGTGTG	*
qPCR			
AR	TTCTGGGTGTCACTATGGAG	ACAAGTTTCTTCAGCTTCC	*
HMOX1	GCAGAGGGTGATAGAAGAGG	AGAATCTTGCACTTTGTTGC	*

* Designed using Primer 3 software: http://frodo.wi.mit.edu/primer3/

### Immunoblotting and Immunofluorescence Staining

Total lysates were analyzed by immunoblotting as described previously [16]. Films were scanned and quantification of the bands was performed using ImageJ software (http://rsbweb.nih.gov/ij/). For immunofluorescence staining, LNCaP-S14 cells were seeded onto 15 mm poly-lysine coated glass cover slips, treated with compounds, and fixed using formaldehyde (3.7% in PBS). Samples were stained with anti-cyclin D1 antibody and nuclei were counterstained with Hoechst dye. Samples were imaged on a Nikon Type 120 inverted fluorescent microscope using 60X magnification.

### Microarray Analysis

Total RNA from cells treated with 40 μM SMIP004, vehicle or positive controls for 24h was obtained using the RNeasy Mini Kit (Qiagen) following the manufacturer's instructions. RNA quality was assessed using a Bioanalyzer (Bio-Rad Experion), and microarray analysis was performed at the Microarray Facility (Sanford-Burnham Medical Research Institute) on the Illumina platform. Briefly, labeled cRNA was generated from total RNA utilizing an RNA amplification kit developed for Illumina (Ambion). First-strand cDNA was synthesized after priming with a T7 promoter-oligo(dT) primer, followed by second strand cDNA synthesis in the presence of biotin-16-UTP. The amplified, biotinylated cRNA was labeled by incubation with streptavidin-Cy3. Hybridization was conducted on the HumanRef-8 v2 Expression BeadChip (Illumina cassettes), which contain ~23,000 human transcript probes and controls. Standard hybridization and wash protocols were used as recommended by the manufacturer. Following high-resolution scanning of the array slides, the BeadStation software was used to deconvolute and present the data in a MIAME compatible tabular form. Several control features are built into the arrays for normalization and quality control. Microarray data has been deposited to NCBI's Gene Expression Omnibus (GEO) repository (GEO Series Accession Number GSE48056).

The averaged expression data for ~15,000 mRNAs for each drug treatment (Supplementary Data File 2) were hierarchically clustered (Spearman Rank Correlation, Complete Linkage Clustering). Pathway analysis was performed using Ingenuity IPA software. A list of 2333 mRNAs that were significantly regulated (≥ 2-fold, p ≤ 0.05) by SMIP004 (Supplementary Data File 3) was imported into IPA and canonical pathways enriched in the dataset were identified. A list of pathways related to stress response, cell cycle, and cancer was selected manually and displayed in the Figure 1.

Network analysis was performed with the Cytoscape Reactome FI plugin [56]. Individual lists of SMIP004-induced and SMIP004-repressed mRNAs (±2-fold, p ≤ 0.05, derived from Supplementary Data File 3) were loaded into Cytoscape and used to build Reactome networks. The networks were clustered into modules, and pathways enriched in the modules (FDR ≤ 0.01) were identified (Fig. 1B).

### Quantitative Proteomic Profiling by Stable Isotope Labeling (SILAC)

LNCaP-S14 cells were metabolically labeled with the heavy amino acids ^13^C_6_ L-lysine and C_6_^15^N_4_-L-arginine (SILAC Metabolic Label, Thermo Scientific) for a period of 6 weeks. “Light” cells maintained in standard media were treated with SMIP004 (40 μM) for 3h, whereas “heavy” cells received vehicle (DMSO, 0.1%). Cells were lysed in a buffer containing 10 mM Tris pH7.4, 0.55% CHAPS, 7.7 M urea, 2.2 M thiourea, 200 mM DTT, 5 μg/ml aprotinin, 10 μg/ml leupeptin, 10 μg/ml pepstatin, 1 mM PMSF. The lysate was rotated 30 minutes at room temperature, and cleared by centrifugation at 15,000 rpm for 15 minutes. Protein concentration was determined by Bradford assay. Cell lysates were mixed at an exact ratio of 1:1. 1.5 mg of mixed lysate was precipitated with methanol and chloroform, and the pellet was dried at room temperature.

SILAC labeled LNCaP-S14 cell lysate pellets were re-suspended in 50 mM ammonium bicarbonate and processed as described before [57]. Briefly, proteins were reduced by TCEP, alkylated using iodoacetamide, and digested by trypsin overnight at 37°C. The peptides were then desalted using Sep-Pak (Millipore), and separated by strong cation chromatography (SCX) using an automated Paradigm HPLC instrument (Michrom Bioresources). The SCX gradient proceeded from 5% – 40% buffer D (25% acetonitrile/0.1% formic acid/ 500 mM KCl), and 24 SCX fractions were collected. Each SCX fraction was analyzed by reversed-phase LC-MS/MS on a Magic C18 column and a LTQ-Orbitrap XL mass spectrometer (Thermo Scientific). The peptides in each SCX fraction were separated on a 60-120 min gradient of 8%-30% buffer B (100% acetonitrile/ 0.1% formic acid). Tandem mass spectrometry (MS/MS) spectra were collected during the LC/MS runs. Each scan was set to acquire a full MS scan, followed by MS/MS scans on the top four precursor ions from the preceding MS scan. The LC-MS/MS analysis of the 24 fractions was repeated 3 times for each biological sample.

Both the protein identification and the SILAC quantification were performed by Integrated Proteomics Pipeline (IP2, Integrated Proteomics Application Inc) which includes SEQUEST, ProLuCID, DTASelect, and Census (The Scripps Research Institute, La Jolla, CA). First, MS1 and MS2 files were extracted by RawExtract (The Scripps Research Institute, La Jolla, CA) from the raw MS/MS spectra obtained on the LTQ-Orbitrap XL. Then an IPI human database was uploaded into IP2, which automatically generated a reverse protein database. The MS1 and MS2 files of each technical replicates were combined and searched using ProLuCID and the semi-tryptic decoy database. Modifications included 16 Da on methionine for oxidation, 6 Da on lysine and 10 Da on arginine for heavy SILAC labels. The identification results from ProLuCID were filtered and organized by DTASelect. The DTASelect parameters were set so that the false positive rates on the protein level were below 1.2%. The false positive rates on the peptide level were < 0.2%. Finally, protein ratios (light/heavy) and the standard deviation were calculated by Census. As expected, the vast majority of proteins showed a ratio very close to 1.0 in the SMIP004-treated versus DMSO-treated cells (Fig. 5A) thus documenting the accuracy of the protein quantification.

Network analysis was performed with the Cytoscape Reactome FI plugin [56]. A list of 580 SMIP004-induced proteins (1.2-fold, p ≤ 0.05) was loaded and used to build a Reactome network, which contained 237 nodes and 1318 edges. The network was clustered into modules, and pathways enriched in the modules (FDR ≤ 0.01) were identified (Supp Fig. 3A).

### Transient Transfection and Luciferase Assay

An AR-luc construct driving the expression of luciferase under the control of the −5400 to +580 region of the androgen receptor promoter [58] was transiently co-transfected with β-gal expression plasmid into LNCaP-S14 cells. Briefly, 1 × 10^5^ LNCaP-S14 cells were seeded in 12 well plates and transfected with 1.6 μg plasmid/well using Lipofectamine 2000 according to the manufacturer's protocol. Six hours after transfection, the medium was changed and the cells were allowed to grow for 24h followed by treatment with SMIP004 or vehicle (DMSO) for another 24h in the presence or absence of R1881 (0.2 nM). Cells were lysed with 150 μl of CCLR reagent (Promega). The chemiluminescence activity in 50 μl of lysate was measured on a Veritas microplate luminometer (Turner Biosystems) and normalized to the β-gal activity.

Plasmids for cyclin D1 (12 μg) and/or CDK4 (12 μg) were transiently transfected into 2 × 10^6^ LNCaP-S14 cells using Lipofectamine 2000. SMIP004 was added 24h after transfection for an additional 24h. Cells were collected for determination of cell cycle distribution by FACS and for immunoblotting analysis.

### Small Interfering (siRNA) Transfection

LNCaP-S14 cells (0.625 × 10^6^ cells) were plated one day prior to transfection. 10-20 nM of siRNA was transfected using DharmaFECT 3 (Thermo Scientific) according to the manufacturer's instructions. Briefly, siRNAs were added to the DharmaFECT 3 reagent pre-diluted in OptiMEM media (1:100) and incubated for 15 min at room temperature. The mixture was added to the cells followed by incubation for 48h. Treatment with SMIP004 or vehicle was carried out for 24h. Cell lysate was obtained and analyzed by immunoblotting. Un-transfected cells as well as cells transfected with non-targeting siRNA were used as controls. siRNAs targeting PERK (siGENOME SMART pool M-004883-03-0005) and IRE1 (siGENOME SMART pool M-004951-02-0005) were purchased from Thermo Scientific. siRNAs targeting CHOP were ordered from Integrated DNA Technologies and had the following sequence: CUCUGUUUCCGUUUCCUGGtt [59]. p27 and p21 siRNAs were as described in [16]. Silencer^®^ Negative Control siRNA #1 from Ambion was used as control siRNA.

### AR mRNA Stability Measurement by Q-PCR

LNCaP-S14 cells were treated with SMIP004 (40 μM) for 9 h followed by addition of actinomycin D (5 μg/ml). Cells were harvested at different time intervals (0, 0.5, 1, 3, 6, 9, 12 and 24h). Total RNA was isolated, followed by first-strand cDNA synthesis using Omniscript^®^ Reverse Transcription kit (Quiagen). Quantitative real-time PCR was performed on the Stratagene Mx3000p detection system (Stratagene, Agilent, CA. USA) using the Brilliant SYBR Green PCR Master Mix (Agilent Technologies). All reactions were done in duplicates.

### PSA Quantitative Determination

LNCaP-S14 cells were plated one day prior to the addition of vehicle, SMIP004 or R1881. Media was collected 24 and 48h after, and PSA secretion was determined using a Human PSA ELISA Kit (Anogen) according to the manufacturer's instructions.

### Metabolic Measurements

For determination of ATP levels, LNCaP-S14 cells were plated in 96 well plates one day prior to addition of SMIP004 (40 μM) in the presence or absence of 10 mM 2-deoxyglucose (2-DG). ATP levels were measured using the ApoSENSOR™ ATP Assay Kit (BioVision). For determination of the GSH/GSSG ratio, LNCaP-S14 cells were treated with SMIP004 (40 μM) for 6h followed by lysis and GSH/GSSG determination using the Glutathione Assay Kit (BioVision).

### Measurement of Oxygen Consumption

Oxygen consumption rate (OCR) was measured in real-time, in an XF24 Extracellular Flux Analyzer (Seahorse Bioscience, Billerica, MA). LNCaP-S14 cells were seeded in XF24-well plates (50000 cells per well in 100 μl). After 6 h, 500 μl media was added followed by incubation overnight at 37°C, 5% CO_2_. The XF24 sensor cartridge was hydrated with 1 ml calibration buffer per well overnight at 37°C. The sensor cartridge was loaded with assay media (ports A, B and C) to measure basal OCR or with oligomycin (1 μM, port A), carbonyl cyanide 4-(trifluoromethoxy)phenylhydrazone (FCCP, 0.5 μM, port B) and rotenone (1 μM, port C) to measure the bioenergetic profile. Cells were washed once with pre-warmed serum-free unbuffered assay medium containing 10 mM sodium pyruvate and 25 mM glucose. 500μl/well of assay medium containing DMSO or SMIP004 (40 μM) was added for measurement of basal OCR. To measure the bioenergetic profile, cells were pre-incubated with DMSO or SMIP004 in regular media for 30, 60 or 120 minutes followed by one wash with assay medium. Cells were kept in 500μl/well of assay medium containing DMSO or SMIP004 (40 μM) thus preserving pre-incubation drug concentrations. Once the sensor cartridge was equilibrated, the calibration plate was replaced with the assay plate. To determine basal OCR and the bioenergetic profile, 12 measurements were taken every 8 minutes.

### Measurement of Mitochondrial Membrane Potential

LNCaP-S14 cells treated with SMIP004 were labeled using tetramethylrhodamine ethyl ester (TMRE, 300 nM) in warm PBS for 30 min followed by trypsinization and two washes with warm PBS containing TMRE (150 nM). Cells were filtered to single suspension and analyzed by flow cytometry using CellQuest (BD Biosciences) and FlowJo (Treestar) software. Cells exposed to the mitochondrial uncoupler carbonyl cyanide *m*-chlorophenyl hydrazone (CCCP, 50 μM) for 45 min were used as positive control.

### Measurement of Reactive Oxygen Species in the Mitochondria

Mitochondrial superoxide production was evaluated using MitoSox™ Red mitochondrial superoxide indicator (Molecular Probes) according to the manufacturer's protocol with some modifications. 3 × 10^5^ cells were seeded into 6 well-plates and allowed to attach for 24h, followed by treatment with SMIP004 (40 μM) for 15, 30, 60 minutes or 3h. Cells were stained with MitoSox (5 μM) for 10 min at 37°C followed by one wash in HBSS buffer. Cells were detached with trypsin and pelleted by centrifugation at 2500 × g for 10 min. The pellet was suspended in 300 μl formaldehyde (3.7%) and incubated for 10 minutes at room temperature, followed by centrifugation at 2500 × g for 10 min. The pellet was suspended in 250 μl HBSS and analyzed by flow cytometry. Measurements were carried out using FACSort and FACSCanto flow cytometers at the Flow Cytometer Facility of the Sanford Burnham Medical Research Institute. MitoSox Red was excited at 488 nm, and data was collected in the 564-606 nm channel.

### LNCaP-S14 Xenograft Studies

Animal experiments were performed in accordance with the institutional animal care committee (IACUC) approved by Sanford Burnham Medical Research Institute, La Jolla. CA. Twelve SCID bg mice (8-10 weeks old) were procured from Harlan Laboratories, Inc., housed under pathogen-free conditions, and maintained on a 12 h light/12 h dark cycle, with food and water supplied ad libidum. LNCaP-S14 cells (5 × 10^6^ cells as a 50% suspension in Matrigel, BD Biosciences, San Jose, CA) in a final volume of 0.4 ml were injected subcutaneously in the flank region of the animals. Tumor size was measured every day until it reached ~ 300 mm^3^. Mice were then injected intraperitoneally (i.p.) with vehicle (six mice, control group) or SMIP004-7 (50 mg/kg) daily (six mice, treated group) for 10 days. Body weights and tumor sizes were measured every other day. Tumor volume was calculated using the equation: volume = length × width × depth × 0.5236 mm^3^. At day 10, three animals per group were sacrificed and tumors were excised and weighed. The remaining six animals (3 control and 3 treated) stayed off treatment for 17 days and were then treated again with vehicle (control) or SMIP004-7 at 100 mg/kg (treated group) for 10 days. At the end of the experiment, animals were sacrificed, tumors weighed, and hematology and blood chemistry analysis were done.

### LAPC4 Xenograft Assay

LAPC4 (10 × 10^6^ cells per animal) were injected into 12 SCID mice as described above. Mice were paired according to their tumor sizes and treated with vehicle or SMIP004-7 (100 mg/kg) for 7 days. Body weights and tumor sizes were recorded daily.

### Breast Cancer Xenografts

All animal experiments were performed in accordance with the institutional animal care committee (IACUC) approved by University of Texas Health Science Center at San Antonio (UTHSCSA). Mice were housed under pathogen-free conditions and maintained on a 12 h light/12 h dark cycle, with food and water supplied ad libidum. Athymic nude mice (Balb c nu/nu, 4-6 weeks old) were procured from Harlan Laboratories (Indianapolis, IN). MDA MB-231 cells (3×10^6^ cells as a 50% suspension in Matrigel, BD Biosciences, San Jose, CA) in a final volume of 0.1 ml were injected subcutaneously in the flank region of Balb c nude mice. Mice were randomized into two groups of 6 mice/group when average tumor volumes reached about 100 mm^3^, and the following treatment protocol was implemented: Group 1, vehicle control administered i.p. for three weeks beginning when tumor volume had reached about 100 mm^3^; Group 2, SMIP004-7 (100 mg/kg body weight) administered ip for three weeks. Body weights and tumor volumes were measured every fifth day. Tumor volumes were calculated using the equation above. After three weeks, mice were sacrificed by cervical dislocation.

## Supplementary Figures and Tables


