# Supplementary material for: Small Molecule-Induced Mitochondrial Disruption Directs Prostate Cancer Inhibition via Unfolded Protein Response Signaling
Source: Oncotarget. 2013 Jul 14;4(8):1212–29. doi: 10.18632/oncotarget.1130 (PMC3787152; doi:10.18632/oncotarget.1130)
Supplement: Supplementary file 1 [file oncotarget-04-1212-s001.pdf]

Supplemental Information

Small Molecule-Mitochondrial Disruption Reprogramming Directs Prostate Cancer Inhibition via Unfolded Protein Response Signaling

Elizabeth Rico-Bautista, Wenhong Zhu, Suthakar Ganapathy, Shinichi Kitada, Eric Lau, Stan Krajewski, Joel Ramirez, Jason A. Bush, Zhimin Yuan, Dieter A. Wolf

- 5 Supplementary Figures
- 1 Supplementary Table

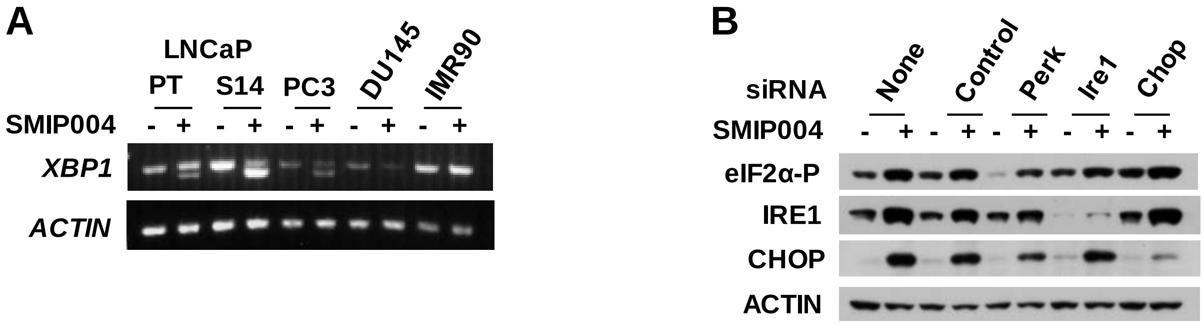

Supplementary Fig 1 (related to Fig. 2). ***XBP1*** **splicing in prostate cancer and normal cells. Knockdown of UPR activators by siRNA transfection.**

(**A**) *XBP1* splicing was evaluated by RT-PCR in total RNA from several prostate cancer cells lines (parental LNCaP-PT, LNCaP-S14, PC3 and DU145) and normal human fibroblast (IMR90) treated with vehicle (DMSO) or SMIP004 (40 μM) for 24 h.

(**B**) Efficiency for siRNA-mediated knockdown of PERK, IRE1 and CHOP was assessed by immunoblotting in cell extracts from LNCaP-S14 cells transfected with the specific siRNAs and followed by treatment with SMIP004 (40 μM) for 24h. PERK knockdown was assessed based on the extent of eIF2α phosphorylation.

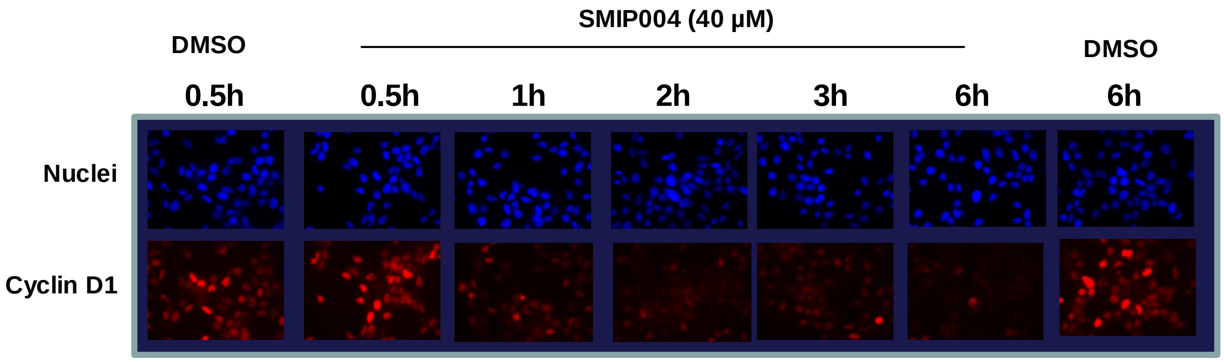

Supplementary Fig 2. (Related to Fig. 3). SMIP004-induced downregulation of cyclin D1

LNCaP-S14 cells were treated with SMIP004 (40 µM) for the indicated times, followed by immunofluorescence staining with cyclin D1 antibodies. Nuclei stained with Hoechst dye are shown for reference.

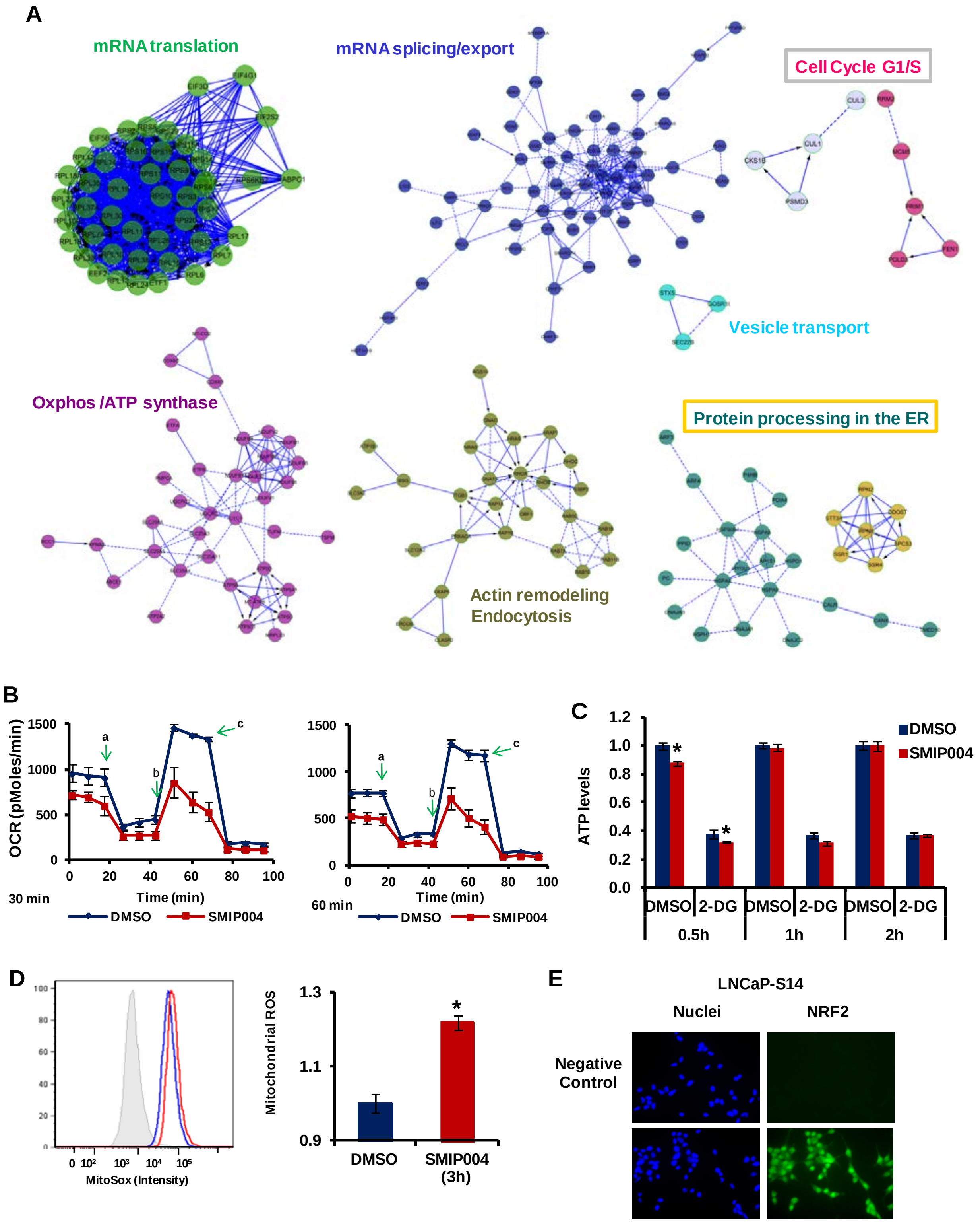

Supplementary Fig 3. (Related to Fig. 5) Effect of SMIP004 on mitochondria and metabolism

(A) Reactome network built from proteins significantly upregulated by SMIP004. The network was clustered into modules, and pathways enriched in the modules (FDR ≤ 0.01) are indicated.

(B) Bioenergetic profile of LNCaP-S14 cells were analyzed in real-time in an XF24 Extracellular Flux Analyzer in the presence or absence of SMIP004. Cells were pre-incubated for 30 min (left graph) or 60 min (right graph) with vehicle (DMSO) or SMIP004 (40µM), followed by real-time analysis of cellular respiration. Oligomycin (a), FCCP (b) and Rotenone (c) were sequentially delivered to the XF-assay medium through injection ports in the sensor cartridge. The graphs represent the mean Oxygen consumption Rate (OCR) ± SEM of three replicates per time point.

(C). LNCaP-S14 cells were treated with SMIP004 (40µM) or DMSO in the presence or absence of 2-deoxyglucose (2-DG) for the indicated time. 2-DG (10 mM) was added 30 min before SMIP004 to inhibit glycolytic ATP production. ATP levels were measured using the ApoSENSOR^{TM} ATP Assay kit (from BioVision). The graph represents the average ± SEM of eight replicates. * p-value < 0.01

(D) Cells were treated with SMIP004 (40 µM) for 3h and mitochondrial superoxide was measured as described in Experimental Procedures. The figure shows representative histograms of flow cytometry experiments demonstrating an increase in mean fluorescence intensity of oxidized MitoSox following SMIP004 treatment (filled grey histogram represents unstained cells). The graph represents the mean fold-induction ± SEM of six replicates.

(E) Basal NRF2 levels were visualized in LNCaP-S14 cells by immunofluorescence staining. Staining with the secondary antibody alone was used as a specificity control.

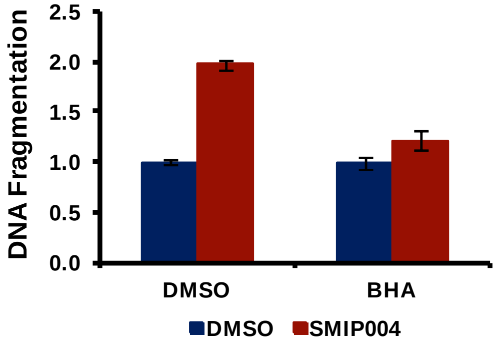

Supplementary Fig 4. (Related to Fig. 6). Effects the antioxidant BHA on SMIP004-dependent apoptosis

DNA fragmentation was measured in extracts from LNCaP-S14 cells treated with SMIP004 (40 µM) or vehicle in the presence or absence of (A) BHA (100 µM, 24h). The graph represents the DNA fragmentation enrichment factor ± standard deviations (normalized to DMSO treatment) of three replicates.

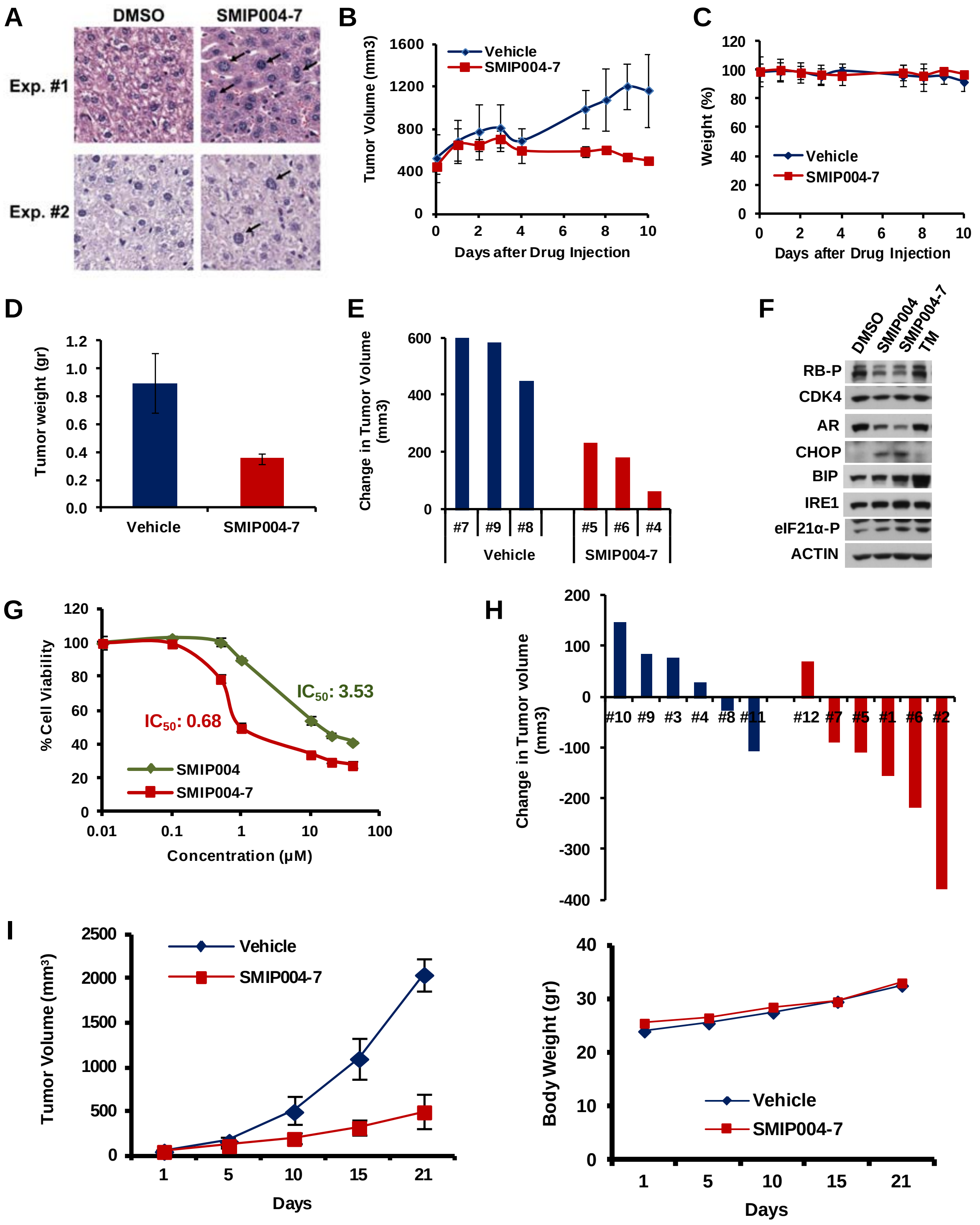

Supplementary Fig 5. (Related to Fig. 7) *In vivo* anti-tumor activity of SMIP004-7

(A) SCID mice were subcutaneously inoculated with LNCaP-S14 cells as described in Experimental Procedures. Animals were treated with SMIP004-7 as described in Fig. 7B. Mice

were sacrificed, liver tissues were fixed, and stained with hematoxylin and eosine prior to histological examination. The arrows highlight cells with enlarged or multiple nuclei.

(B) At the end of the treatment period, compound treatment of three mice was suspended for 17 days. During the suspension period, the tumors resumed growth indicating that the cells originally received by the mice were competent of forming tumors. When tumors had reached ~500 mm^3, the pretreated animals were subjected to a second period of treatment with an increased dose of SMIP007-4 (100 mg/kg) daily for 10 days. The graph shows changes in tumor volumes over time. As in the first treatment period (Fig. 7B), SMIP004-7 receiving animals showed a strong inhibition in tumor growth whereas tumors grew to large sizes in vehicle treated mice.

(C) Average of body weights ± standard deviations normalized to the body weight at day zero of treatment.

(D) Final tumor weight of mice treated with vehicle or SMIP004-7. The graph represents the average of three mice ± standard deviations.

(E) The response to SMIP004-7 or vehicle for each animal was expressed as the change in tumor volume (tumor size day eight – tumor size day zero).

(F) Total extracts from LAPC4 treated with SMIP004 (40 µM), SMIP004-7 (40 µM), or tunicamycin (1 µM) were obtained and analyzed by immunoblotting for cell cycle and ER stress markers.

(G) LAPC4 cells were treated with increasing concentrations of SMIP004 or SMIP004-7 (0.1 µM to 40 µM) for 72 hours, and cell viability was measured by MTT assay. The graph represents the mean ± standard deviations of three replicates per point (normalized to untreated cells).

(H) LAPC4 xenografts were performed as described in Supplementary Experimental Procedures, and changes in tumor volumes (mm^3) (day 7 – day 0) were calculated for each animal.

(I) MDA-MB 231 xenografts were performed as described in the Supplementary Experimental Procedures. The graph to the left represents the tumor volume ± standard deviations over time. The graph to the right represents changes in body weights (average ± standard deviations).

Supplementary Table 1 Blood chemistry of SMIP004-7-treated mice

		Control Group					SMIP004-7 group					
**Hematology**	**Unit**	**Mouse#7**	**Mouse#8**	**Mouse#9**	**Mean**	**SD**	**Mouse#1**	**Mouse#2**	**Mouse#3**	**Mean**	**SD**	**Normal Range**
White Blood Cells	x 10^9/l	2.06	1.71	1.36	**1.71**	**0.35**	2.69	2.27	2.76	**2.57**	**0.27**	6.0-15.0
Lymphocytes	x 10^9/l	0.28	0.17	0.36	**0.27**	**0.10**	0.24	0.32	0.94	**0.50**	**0.38**	3.4-7.44
Monocytes	x 10^9/l	0.23	0.14	0.19	**0.19**	**0.05**	0.28	0.27	0.21	**0.25**	**0.04**	< 0.6
Granulocyte	x 10^9/l	1.56	1.41	0.81	**1.26**	**0.40**	2.18	1.69	1.61	**1.83**	**0.31**	0.5-3.8
Lymphocytes %	%	13.4	10	26.8	**16.73**	**8.88**	8.8	14	34.1	**18.97**	**13.36**	57-93
Monocytes %	%	11.1	7.9	13.8	**10.93**	**2.95**	10.3	11.8	7.7	**9.93**	**2.07**	<7
Granulocytes %	%	75.5	82.1	59.4	**72.33**	**11.68**	80.9	74.2	58.2	**71.10**	**11.66**	8.0-48
Red Blood Cells	x10^12/l	8.77	8.01	8.17	**8.32**	**0.40**	6.2	6.35	7.23	**6.59**	**0.56**	7.0-12
Hemoglobin	g/dl	14.1	12.2	12.9	**13.07**	**0.96**	14.7	16.2	18.3	**16.40**	**1.81**	12.2-16.2
Hematocrite	%	39.48	36.18	36.74	**37.47**	**1.77**	35.24	33.69	36.62	**35.18**	**1.47**	35-45
Platelet	x 10^9/l	907	680	636	**741.00**	**145.43**	913	864	859	**878.67**	**29.84**	200-450
**Chemistry**		**Mouse#7**	**Mouse#8**	**Mouse#9**	**Mean**	**SD**	**Mouse#1**	**Mouse#2**	**Mouse#3**	**Mean**	**SD**	**Normal Range**
Albumin	g/dl	2.5	3.5	3.7	**3.23**	**0.64**	3.3	2.4	3.1	**2.93**	**0.47**	2.5-3.0
Alkaline Phosphatase	U/L	18	64	69	**50.33**	**28.11**	19	31	14	**21.33**	**8.74**	35-98
Alanine Aminotransferase	U/L	110	90	54	**84.67**	**28.38**	87	27	36	**50.00**	**32.36**	17-77
Amylase	U/L	770	842	827	**813.00**	**37.99**	880	713	864	**819.00**	**92.15**	
Total Bilirubin	mg/dl	0.2	0.2	0.2	**0.20**	**0.00**	0.2	0.3	0.2	**0.23**	**0.06**	0.0-0.9
Blood Urea Nitrogen	mg/dl	19	19	17	**18.33**	**1.15**	25	18	18	**20.33**	**4.04**	8.0-33
Calcium	mg/dl	9.8	10.3	10.4	**10.17**	**0.32**	9.1	8.7	9.5	**9.10**	**0.40**	7.1-10.1
Phosphorus	mg/dl	7.8	5.8	5.2	**6.27**	**1.36**	9.2	7.3	8	**8.17**	**0.96**	5.7-9.2
Creatinine	mg/dl	0.1	0.2	0.2	**0.17**	**0.06**	0.2	0.2	0.2	**0.20**	**0.00**	0.2-0.9
Glucose	mg/dl	188	295	249	**244.00**	**53.67**	171	160	291	**207.33**	**72.67**	62-175
Sodium	mM	150	152	155	**152.33**	**2.52**	150	147	149	**148.67**	**1.53**	140-160
Potassium	mM	9.9	7.2	8.2	**8.43**	**1.37**	7.5	6.9	7.6	**7.33**	**0.38**	5.0-7.5
Total Protein	g/dl	5.7	5.9	5.6	**5.73**	**0.15**	5.2	4.8	4.5	**4.83**	**0.35**	3.5-7.2
Globulin	g/dl	1.9	2.4	1.8	**2.03**	**0.32**	1.9	2.4	1.4	**1.90**	**0.50**	1.0-4.2
